# {4,4′,6,6′-Tetra­iodo-2,2′-[propane-1,3-diylbis(nitrilo­methanylyl­idene)]diphenolato-κ^4^ *O*,*N*,*N*′,*O*′}nickel(II)

**DOI:** 10.1107/S1600536812032138

**Published:** 2012-07-18

**Authors:** Hadi Kargar, Reza Kia, Amir Adabi Ardakani, Muhammad Nawaz Tahir

**Affiliations:** a Department of Chemistry, Payame Noor University, PO Box 19395-3697 Tehran, I. R. of IRAN; b Department of Chemistry, Science and Research Branch, Islamic Azad University, Tehran, Iran; c Ardakan Branch, Islamic Azad University, Ardakan, Iran; d Department of Physics, University of Sargodha, Punjab, Pakistan

## Abstract

The asymmetric unit of the title compound, [Ni(C_17_H_12_I_4_N_2_O_2_)], comprises half of a Schiff base complex. The Ni^II^ and central C atom of the propyl chain are located on a twofold rotation axis. The geometry around the Ni^II^ atom is square planar, supported by the N_2_O_2_ donor atoms of the coordinated ligand. In the crystal, there are no significant inter­molecular inter­actions present. The crystal studied was a non-merohedral twin with a refined twin component ratio of 0.944 (1):0.056 (1).

## Related literature

For standard bond lengths, see: Allen *et al.* (1987 ▶). For applications of Schiff bases in coordination chemistry, see, for example: Granovski *et al.* (1993 ▶); Blower *et al.* (1998 ▶). For the structure of the Schiff base ligand, see: Kargar *et al.* (2012*a* ▶). For related structures, see, for example: Kargar *et al.* (2012*b* ▶,*c* ▶,*d* ▶,*e* ▶).
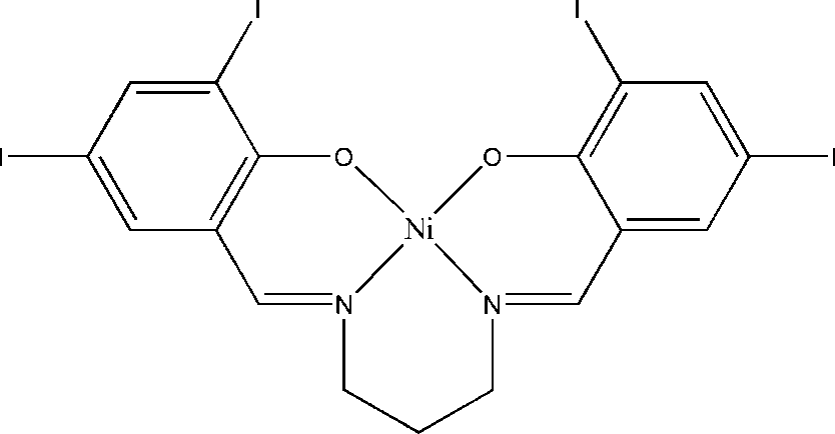


## Experimental

### 

#### Crystal data

[Ni(C_17_H_12_I_4_N_2_O_2_)]
*M* *_r_* = 842.60
Monoclinic, 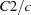

*a* = 26.1229 (18) Å
*b* = 10.7409 (7) Å
*c* = 7.2387 (5) Å
β = 98.107 (3)°
*V* = 2010.8 (2) Å^3^
*Z* = 4
Mo *K*α radiation
μ = 7.12 mm^−1^
*T* = 291 K
0.22 × 0.12 × 0.08 mm

#### Data collection

Bruker SMART APEXII CCD area-detector diffractometer
Absorption correction: multi-scan (*TWINABS*; Bruker, 2005 ▶) *T*_min_ = 0.303, *T*_max_ = 0.600
7468 measured reflections
2188 independent reflections
1755 reflections with *I* > 2σ(*I*)
*R*_int_ = 0.033

#### Refinement

*R*[*F* ^2^ > 2σ(*F* ^2^)] = 0.031
*wR*(*F* ^2^) = 0.055
*S* = 1.04
2188 reflections
120 parameters
H-atom parameters constrained
Δρ_max_ = 0.70 e Å^−3^
Δρ_min_ = −0.55 e Å^−3^

### 

Data collection: *APEX2* (Bruker, 2005 ▶); cell refinement: *SAINT* (Bruker, 2005 ▶); data reduction: *SAINT*; program(s) used to solve structure: *SHELXS97* (Sheldrick, 2008 ▶); program(s) used to refine structure: *SHELXL97* (Sheldrick, 2008 ▶); molecular graphics: *SHELXTL* (Sheldrick, 2008 ▶); software used to prepare material for publication: *SHELXTL* and *PLATON* (Spek, 2009 ▶).

## Supplementary Material

Crystal structure: contains datablock(s) global, I. DOI: 10.1107/S1600536812032138/su2472sup1.cif

Structure factors: contains datablock(s) I. DOI: 10.1107/S1600536812032138/su2472Isup2.hkl

Additional supplementary materials:  crystallographic information; 3D view; checkCIF report

## Figures and Tables

**Fig. 1.**
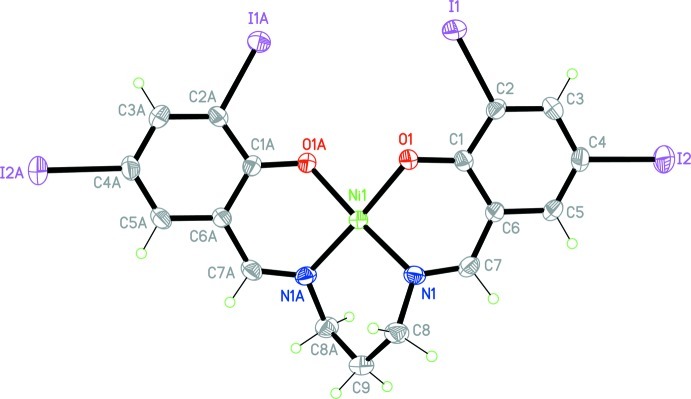
A view of the molecular structure of the title molecule, showing 50% probability displacement ellipsoids and the atomic numbering [symmetry code for suffix A = -x, y, -z + 1/2].

## supplementary crystallographic information

### Comment

Schiff base complexes are one of the most important stereochemical models in transition metal coordination chemistry, with their ease of preparation and structural variations (Granovski *et al.*, 1993; Blower *et al.*, 1998). In continuation of our work on the crystal structure of Schiff base metal complexes (Kargar *et al.*, 2012**b*,*c*,*d*,e*), we determined the X-ray structure of the title compound.

The asymmetric unit of the title compound, Fig. 1, comprises a Schiff base complex. The bond lengths (Allen *et al.*, 1987) and angles are within the normal ranges and are comparable to the bond lengths and angles of the related ligand (Kargar *et al.*, 2012*a*) and related Ni-complexes (Kargar *et al.*, 2012**b*,*c*,*d*,e*). The Ni^II^ and C9 atom of the propyl segment are located on a two-fold rotation axis. The geometry around Ni^II^ atom is square-planar which is supported by the N_2_O_2_ donor atoms of the coordinated ligand.

There are no significant intermolecular interactions in the crystal structure.

The crystal used was a non-merohedral twin with refined twin components ratio of 0.944 (1)/0.056 (1).

### Experimental

The title compound was synthesized by adding 3,5-diiodo-salicylaldehyde-1,3-propanediamine (2 mmol) to a solution of NiCl_2_. 6H_2_O (2.1 mmol) in ethanol (30 ml). The mixture was refluxed with stirring for 1h. The resultant solution was filtered. Red single crystals of the title compound suitable for *X*-ray structure determination were recrystallized from ethanol by slow evaporation of the solvents at room temperature over several days.

### Refinement

The H-atoms were included in calculated positions and treated as riding atoms: C—H = 0.93 and 0.97 Å for CH and CH_2_ H-atoms, respectively, with U_iso_ (H) = 1.2 U_eq_(C).

### Crystal data

**Table:**

[Ni(C_17_H_12_I_4_N_2_O_2_)]	*F*(000) = 1536
*M**_r_* = 842.60	*D*_x_ = 2.783 Mg m^−^^3^
Monoclinic, *C*2/*c*	Mo *K*α radiation, λ = 0.71073 Å
Hall symbol: -C 2yc	Cell parameters from 526 reflections
*a* = 26.1229 (18) Å	θ = 2.5–27.5°
*b* = 10.7409 (7) Å	µ = 7.12 mm^−^^1^
*c* = 7.2387 (5) Å	*T* = 291 K
β = 98.107 (3)°	Block, red
*V* = 2010.8 (2) Å^3^	0.22 × 0.12 × 0.08 mm
*Z* = 4	

### Data collection

**Table:**

Bruker SMART APEXII CCD area-detector diffractometer	2188 independent reflections
Radiation source: fine-focus sealed tube	1755 reflections with *I* > 2σ(*I*)
Graphite monochromator	*R*_int_ = 0.033
ω scans	θ_max_ = 27.1°, θ_min_ = 1.6°
Absorption correction: multi-scan (*TWINABS*; Bruker, 2005)	*h* = −33→33
*T*_min_ = 0.303, *T*_max_ = 0.600	*k* = −13→13
7463 measured reflections	*l* = −8→9

### Refinement

**Table:**

Refinement on *F*^2^	Primary atom site location: structure-invariant direct methods
Least-squares matrix: full	Secondary atom site location: difference Fourier map
*R*[*F*^2^ > 2σ(*F*^2^)] = 0.031	Hydrogen site location: inferred from neighbouring sites
*wR*(*F*^2^) = 0.055	H-atom parameters constrained
*S* = 1.04	*w* = 1/[σ^2^(*F*_o_^2^) + (0.0177*P*)^2^ + 2.2669*P*] where *P* = (*F*_o_^2^ + 2*F*_c_^2^)/3
2188 reflections	(Δ/σ)_max_ = 0.001
120 parameters	Δρ_max_ = 0.70 e Å^−^^3^
0 restraints	Δρ_min_ = −0.55 e Å^−^^3^

### Special details

**Table:**

Geometry. All esds (except the esd in the dihedral angle between two l.s. planes) are estimated using the full covariance matrix. The cell esds are taken into account individually in the estimation of esds in distances, angles and torsion angles; correlations between esds in cell parameters are only used when they are defined by crystal symmetry. An approximate (isotropic) treatment of cell esds is used for estimating esds involving l.s. planes.
Refinement. Refinement of F^2^ against ALL reflections. The weighted R-factor wR and goodness of fit S are based on F^2^, conventional R-factors R are based on F, with F set to zero for negative F^2^. The threshold expression of F^2^ > 2sigma(F^2^) is used only for calculating R-factors(gt) etc. and is not relevant to the choice of reflections for refinement. R-factors based on F^2^ are statistically about twice as large as those based on F, and R- factors based on ALL data will be even larger.

### Fractional atomic coordinates and isotropic or equivalent isotropic displacement parameters (Å^2^)

**Table:**

	*x*	*y*	*z*	*U*_iso_*/*U*_eq_	Occ. (<1)
Ni1	0.0000	0.48944 (7)	0.2500	0.02132 (18)	
I1	0.090843 (12)	0.89044 (3)	0.36252 (5)	0.03652 (10)	
I2	0.279631 (12)	0.60955 (3)	0.20407 (5)	0.04421 (11)	
N1	0.05062 (13)	0.3679 (3)	0.3138 (5)	0.0242 (8)	
O1	0.04733 (11)	0.6174 (2)	0.3090 (4)	0.0269 (7)	
C1	0.09612 (15)	0.6129 (4)	0.2932 (6)	0.0224 (9)	
C2	0.12636 (16)	0.7229 (4)	0.3024 (6)	0.0240 (9)	
C3	0.17729 (16)	0.7234 (4)	0.2769 (6)	0.0291 (10)	
H3	0.1954	0.7980	0.2804	0.035*	
C4	0.20183 (16)	0.6119 (4)	0.2457 (6)	0.0291 (10)	
C5	0.17586 (16)	0.5018 (4)	0.2474 (6)	0.0276 (10)	
H5	0.1930	0.4273	0.2327	0.033*	
C6	0.12328 (15)	0.5005 (4)	0.2715 (6)	0.0233 (10)	
C7	0.09902 (16)	0.3837 (4)	0.3042 (6)	0.0271 (10)	
H7	0.1201	0.3136	0.3198	0.033*	
C8	0.03503 (17)	0.2492 (4)	0.3903 (6)	0.0292 (10)	
H8A	0.0173	0.2661	0.4967	0.035*	
H8B	0.0658	0.2012	0.4344	0.035*	
C9	0.0000	0.1721 (5)	0.2500	0.0298 (14)	
H9A	−0.0212	0.1188	0.3165	0.036*	0.50
H9B	0.0212	0.1188	0.1835	0.036*	0.50

### Atomic displacement parameters (Å^2^)

**Table:**

	*U*^11^	*U*^22^	*U*^33^	*U*^12^	*U*^13^	*U*^23^
Ni1	0.0199 (4)	0.0179 (4)	0.0259 (4)	0.000	0.0026 (3)	0.000
I1	0.03286 (18)	0.02414 (16)	0.0539 (2)	0.00044 (13)	0.01095 (15)	−0.00832 (15)
I2	0.02416 (17)	0.0449 (2)	0.0661 (3)	0.00048 (15)	0.01512 (16)	0.00269 (17)
N1	0.0261 (19)	0.0191 (19)	0.026 (2)	−0.0019 (15)	0.0007 (16)	0.0025 (15)
O1	0.0204 (15)	0.0220 (15)	0.0392 (19)	−0.0010 (12)	0.0072 (13)	−0.0029 (13)
C1	0.021 (2)	0.024 (2)	0.022 (2)	−0.0018 (18)	0.0026 (18)	0.0021 (18)
C2	0.023 (2)	0.024 (2)	0.025 (2)	0.0037 (18)	0.0038 (19)	−0.0022 (18)
C3	0.025 (2)	0.029 (2)	0.033 (3)	−0.007 (2)	0.003 (2)	−0.002 (2)
C4	0.019 (2)	0.034 (3)	0.034 (3)	0.002 (2)	0.0038 (19)	0.000 (2)
C5	0.024 (2)	0.025 (2)	0.033 (3)	0.0070 (19)	0.004 (2)	−0.0007 (19)
C6	0.021 (2)	0.022 (2)	0.027 (2)	0.0014 (18)	0.0023 (19)	−0.0017 (18)
C7	0.025 (2)	0.024 (2)	0.031 (3)	0.0064 (19)	0.001 (2)	0.0020 (19)
C8	0.032 (2)	0.025 (2)	0.030 (3)	0.000 (2)	0.000 (2)	0.0072 (19)
C9	0.036 (4)	0.021 (3)	0.033 (4)	0.000	0.005 (3)	0.000

### Geometric parameters (Å, º)

**Table:**

Ni1—O1^i^	1.858 (3)	C3—H3	0.9300
Ni1—O1	1.858 (3)	C4—C5	1.365 (6)
Ni1—N1^i^	1.869 (3)	C5—C6	1.409 (6)
Ni1—N1	1.869 (3)	C5—H5	0.9300
I1—C2	2.098 (4)	C6—C7	1.440 (5)
I2—C4	2.096 (4)	C7—H7	0.9300
N1—C7	1.287 (5)	C8—C9	1.514 (5)
N1—C8	1.471 (5)	C8—H8A	0.9700
O1—C1	1.296 (5)	C8—H8B	0.9700
C1—C2	1.418 (5)	C9—C8^i^	1.514 (5)
C1—C6	1.421 (5)	C9—H9A	0.9700
C2—C3	1.369 (5)	C9—H9B	0.9700
C3—C4	1.391 (6)		
			
O1^i^—Ni1—O1	84.57 (17)	C4—C5—C6	120.3 (4)
O1^i^—Ni1—N1^i^	92.01 (13)	C4—C5—H5	119.8
O1—Ni1—N1^i^	176.57 (13)	C6—C5—H5	119.8
O1^i^—Ni1—N1	176.57 (13)	C5—C6—C1	121.1 (4)
O1—Ni1—N1	92.01 (13)	C5—C6—C7	119.3 (4)
N1^i^—Ni1—N1	91.4 (2)	C1—C6—C7	118.9 (4)
C7—N1—C8	117.4 (3)	N1—C7—C6	125.6 (4)
C7—N1—Ni1	124.1 (3)	N1—C7—H7	117.2
C8—N1—Ni1	118.3 (3)	C6—C7—H7	117.2
C1—O1—Ni1	125.6 (3)	N1—C8—C9	113.2 (4)
O1—C1—C2	120.9 (4)	N1—C8—H8A	108.9
O1—C1—C6	123.7 (4)	C9—C8—H8A	108.9
C2—C1—C6	115.5 (4)	N1—C8—H8B	108.9
C3—C2—C1	122.9 (4)	C9—C8—H8B	108.9
C3—C2—I1	119.4 (3)	H8A—C8—H8B	107.7
C1—C2—I1	117.7 (3)	C8—C9—C8^i^	113.7 (5)
C2—C3—C4	119.8 (4)	C8—C9—H9A	108.8
C2—C3—H3	120.1	C8^i^—C9—H9A	108.8
C4—C3—H3	120.1	C8—C9—H9B	108.8
C5—C4—C3	120.2 (4)	C8^i^—C9—H9B	108.8
C5—C4—I2	118.9 (3)	H9A—C9—H9B	107.7
C3—C4—I2	120.8 (3)		
			
N1^i^—Ni1—N1—C7	152.0 (4)	I2—C4—C5—C6	−178.8 (3)
N1^i^—Ni1—N1—C8	−31.8 (2)	C4—C5—C6—C1	0.4 (6)
O1^i^—Ni1—O1—C1	−148.9 (4)	C4—C5—C6—C7	−169.6 (4)
N1—Ni1—O1—C1	31.2 (3)	O1—C1—C6—C5	177.4 (4)
Ni1—O1—C1—C2	166.0 (3)	C2—C1—C6—C5	−4.4 (6)
Ni1—O1—C1—C6	−15.9 (6)	O1—C1—C6—C7	−12.5 (6)
O1—C1—C2—C3	−176.6 (4)	C2—C1—C6—C7	165.7 (4)
C6—C1—C2—C3	5.2 (6)	C8—N1—C7—C6	−166.2 (4)
O1—C1—C2—I1	4.6 (5)	Ni1—N1—C7—C6	10.0 (6)
C6—C1—C2—I1	−173.7 (3)	C5—C6—C7—N1	−174.2 (4)
C1—C2—C3—C4	−1.9 (7)	C1—C6—C7—N1	15.6 (7)
I1—C2—C3—C4	177.0 (3)	C7—N1—C8—C9	−116.1 (4)
C2—C3—C4—C5	−2.4 (7)	Ni1—N1—C8—C9	67.4 (4)
C2—C3—C4—I2	179.5 (3)	N1—C8—C9—C8^i^	−32.1 (2)
C3—C4—C5—C6	3.2 (7)		

Symmetry code: (i) −*x*, *y*, −*z*+1/2.
